# Synthesis, optical imaging, and absorption spectroscopy data for 179072 metal oxides

**DOI:** 10.1038/s41597-019-0019-4

**Published:** 2019-03-27

**Authors:** Helge S. Stein, Edwin Soedarmadji, Paul F. Newhouse, Dan Guevarra, John M. Gregoire

**Affiliations:** Joint Center for Artificial Photosynthesis, California Institute of Technology, Pasadena, California 91125 USA

## Abstract

Optical absorption spectroscopy is an important materials characterization for applications such as solar energy generation. This data descriptor describes the to date (Dec 2018) largest publicly available curated materials science dataset for near infrared to near UV (UV-Vis) light absorbance, composition and processing properties of metal oxides. By supplying the complete synthesis and processing history of each of the 179072 samples from 99965 unique compositions we believe the dataset will enable the community to develop predictive models for materials, such as prediction of optical properties based on composition and processing, and ultimately serve as a benchmark dataset for continued integration of machine learning in materials science. The dataset is also a resource for identifying materials composition and synthesis to attain specific optical properties.

## Abstract (summary)

**Table:** 

Design Type(s)	classification objective • observation design
Measurement Type(s)	absorption spectrum
Technology Type(s)	ultraviolet-visible spectrophotometry
Factor Type(s)	metal oxide
Sample Characteristic(s)

Machine-accessible metadata file describing the reported data (ISA-Tab format)

## Background & Summary

The availability of scientific database systems^1^, fast measurement instruments^2^ and network infrastructures enable scientists to assemble ultra large datasets that enable to go beyond the answering of some original research question and gain fundamentally new knowledge via learning on all data collected^3^. Currently, fields such as organic chemistry^4^, drug design^5–7^, ab-initio materials science^8^, and biology gain rapid pace through the availability of large datasets that enable predictive machine learning models but experimental materials science lacks such ultra large datasets (with the notable exception of the High-throughput Experimental Materials Database - HTEM^1^) as different synthesis procedures, processing conditions and analyses effectively block the assembly due to prohibitive inconsistencies in the data across experimental runs. Within the Joint Center for Artificial Photosynthesis, exploration of metal oxides for solar fuels generation included high throughput synthesis and optical characterization with tracking of synthesis and processing parameters. The exploration of the chemical space offered by the periodic table was not randomly or systematically explored as compositions spaces were chosen based on specific research directions.

Recently we published an algorithm paper that allows us to predict UV-Vis data based on a sample image^9^ via a neural net machine learning model that effectively hyper scales the low energy resolution RGB image to optical absorbance values at 220 energies between 1.32 to 3.2 eV. The herewith published dataset contains all images and spectra used for this model.

This dataset^10^ will enable materials scientists to continue developing algorithms that build upon recent advances including finding embeddings for materials composition^11,12^, predicting optical properties^9^ from composition, linking experimental findings to theory databases^8,13^, and extracting band gap energy from UV-Vis spectra^14,15^.

By making the dataset available as a hdf5^16^ container we aim to make the dataset more amendable for scientists who are not fluent in database query languages as all data is organized in tabular format where every entry corresponds to the same sample. In this manuscript we will give some background about how the dataset was acquired and is structured.

## Methods

These methods are expanded versions of descriptions in our related work, which is referenced below for each technique. All samples in this dataset were synthesized via ink-jet printing of precursor salts with subsequent thermal processing to form metal oxides^17^. Mostly this synthesis involves printing metal nitrate salts on a glucose coated FTO/Glass substrate. The general assumption is that any chosen metal precursor salt, e.g. Mn(NO_3_)_2_, will thermally decompose under oxidizing conditions into a metal oxide, e.g. Mn oxide, via removal of the precursor’s anion as a gas, e.g. NO_2_. A typical thermal processing is annealing at 500 °C for 1 h in air or synthetic air. Some compositions, especially pure elemental oxides, are duplicated many times in the dataset, which can be readily identified via the composition table.

### Sample image generation

All sample images were taken using a commercially-available consumer flatbed scanner (EPSON Perfection V600) in reflection configuration at 1200 dpi corresponding to a rate of 2.0 cm^2^ s^−1^ or 0.019 s per sample as described elsewhere^18^. We assumed no lamp drift over time as the scanner is equipped with LED light sources. The scanner takes an images of a complete plate that is diced into 2.1 mm × 2.1 mm or 101 × 101 pixels with 24 bit color depth. Dicing of images was done semi automatically as scientists told the algorithm where fiducials for alignment were subsequent to scanning. To reduce the data size all images were rescaled to 64 × 64 pixels via the python image library (pillow) with anti-aliasing. Sample images typically have a colored region in the center corresponding to the printed material surrounded by grey area that is the background signal of the glass in the scanner bed. Some images appear darker at the edge of the printed material due to the so-called coffee ring that forms during drying of the printed solutions.

### UV-Vis spectra measurement

All optical absorption spectra were measured using an on-the-fly scanning UV-Vis dual-sphere spectrometer as described elsewhere^19^. Since the spectral range over which the data was acquired varied, we interpolated on the smallest common energy range, 1.31 to 3.1 eV, which we discretize into 220 photon energies. We report fractional optical absorbance, which is the product of the absorption coefficient *α* and effective material thickness *L*, calculated via measurements of the fractional total reflectance R and total transmittance T:

$$\alpha L=-ln\frac{T}{1-R}.$$

### Composition calculation

All samples are labelled with their intended metals composition. Various quality control methods, which are not annotated in the dataset, were employed to omit samples whose composition is believed to differ from the intended composition. These methods include optical inspection and X-ray fluorescence measurements of the elemental loadings. The oxygen concentration results from thermal processing and is unknown. To enable researchers to study thickness effects of materials the loading as well as atomic fractions are reported. The total loading is the sum of loadings for each sample from which the atomic fractions were calculated. Loadings are calculated from ink concentration and known deposited volumes.

## Code Availability

Custom code for handling the dataset is available at https://github.com/helgestein/materials-images-spectra/. This python code enables users to easily download the dataset, pull specific or random images and accompanying spectra as well as processing and composition data. The code is intended to enable easy exploration of the dataset and to provide templates for use in machine learning models. The code requires python version 3.6.4 or higher with the following packages: h5py > = 2.7.1, numpy >  = 1.15.2, tqdm >  = 4.23.0.

## Data Records

**Fig. 1:**
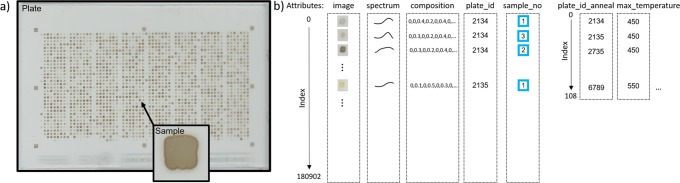
Data layout comparison between plate and data container. The logical layout is shown in (**a**) and the hdf5 container layout is shown in (**b**). Each plate can contain one or multiple composition spaces where each sample is uniquely defined by its sample ID, and plate ID pair. The logical layout is flattened in the hdf5 container such that all samples are placed along a single index.

During preparation of the hdf5 container we used the h5py library version 2.7.1 on a Windows 10 workstation. Images and spectra are compressed using the gzip option during creation of the file. The container has several attributes (see Fig. 1) that will be briefly described and are summarized in Table 1. The largest attribute in terms of data amount is the images that are 64 × 64 pixel containing each 3 colors corresponding to red, green, blue. All color values are floating point values between 0 and 1. In the spectrum dataset all spectra are placed in the same order as images. The composition of each sample is stored in the composition dataset as an array of concentrations for 42 elements in the dataset (most concentration values are zero). It should be noted that not all compositions sum to unity due to rounding error. The element labels (loadings and normalized atomic fractions) are stored separately as a string dataset in the “loadings” and “atfrac” datasets. The loading array contains 1 additional dimension for the total loading. Tracking indices for each library plate and each sample within a plate are stored in the correspondingly named attributes. Other information such as the anneal conditions are described in the last 5 rows of Table 1.

**Table 1:** Summary of all attributes in the hdf5 container accompanying this manuscript. All attributes contain arrays of the tuple shape given in the data size column.

Dataset	Content Description	Data Range	Data Size	Physical Units	Method
Images	Sample images	0–1 for every channel	(64,64,3,180902)	Color values for RGB	platebead scanner
spectra	fractional optical absorbance spectrum	0–ca. 0.5	(220,180902)	fractional absorb. coefficient	dual-sphere optical spectrometer
loadings	loading of each element	0–1	(43,180902)	nmol	calculated from loading and ink concentration
atfrac	Atomic fractions	0–1	(42,180902)	fractions	calculated from loadings
plate_id	Identifier index for plate	integer	(1,180902)	none	assigned
sample_id	Identifier index for each sample	integer	(1,180902)	none	assigned
energy_eV	Energy axis for spectra	float	(220,1)	Electron Volt (eV)	measured by spectrometer
loading_keys	Identifier index for loading element	String starting with Element	String list 180902 entries	Element names	assigned
atfrac_keys	Identifier index for loading element	String starting with Element	String list 180902 entries	Element names	assigned
substrate	Substrate used	string	String list 108 entries	none	assigned
plate_id_anneal	Maximum temperature during anneal	integer	(1,108)	none	assigned
max_temperature	Maximum temperature the plate was annealed at	float	(1,108)	Celcius	anneal recipe
soak_time_at_max_temperature	Time at maximum temperature	float	(1,108)	minutes	anneal recipe
nominal_pressure	Nominal pressure at maximum temperature	float	(1,108)	Torr	anneal recipe
gas_composition_string	Composition of the annealing gas	string	108 Strings	none	anneal recipe
intended_element	Element intended to be added during anneal	string	108 Strings	none	anneal recipe

There are 180902 discrete samples, 1830 of which are “reference” samples where no material was deposited on the substrate, leaving 179072 materials samples. Due to duplication of compositions to enable exploration of different synthesis conditions, provide internal standards, and evaluate reproducibility, various compositions appear multiple times in the database, sometimes with variation in the synthesis conditions. Rounding to the nearest 1 at.% (although composition intervals are typically 5 at.%), there are 99965 unique compositions. The total number of plates is 108, each containing about 2000 samples.

## Technical Validation

Each sample in the dataset is part of a library plate that was visually inspected for printing quality during the materials synthesis phase. Detailed validation of the composition and other properties of individual samples have been performed on a small subset of the samples, with the only present availability of this data being journal publications describing specific libraries^14,18,20–22^. The array of materials in a library plate are indexed with sample location determined in each measurement using printed fiducials.

Standard data analysis software like the open source hdf5 library for python (https://www.h5py.org/) can read the container.

**Fig. 2:**
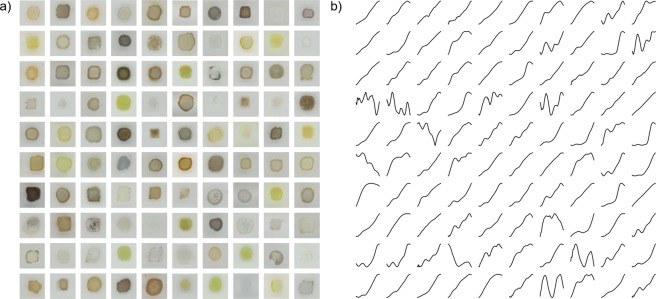
Comparison of materials images and their spectra. (**a**) Example images from the dataset with their corresponding (**b**) fractional optical absorbance spectra. The energy range for all spectra is 1.32 eV (left end) to 3.1 eV (right end).

Example images and corresponding spectra are shown in Fig. 2.

## ISA-Tab metadata file

Download metadata file
